# The effect of age on outpatient pediatric procedural sedation with intranasal dexmedetomidine and oral midazolam

**DOI:** 10.1007/s00431-023-05240-5

**Published:** 2023-10-19

**Authors:** Xiaqing Zhou, Jialian Zhao, Haiya Tu, Kunwei Chen, Yaoqin Hu, Yue Jin

**Affiliations:** Department of Anesthesiology, the Children’s Hospital, Zhejiang University School of Medicine, Hangzhou, Zhejiang China

**Keywords:** Dexmedetomidine, Midazolam, Procedural sedation, Pediatric, Age

## Abstract

Procedural sedation for diagnostic examination is a common practice in children. The study aims to analyze the sedative effect and safety of intranasal dexmedetomidine combined with oral midazolam in outpatient pediatric procedural sedation across different age groups and to assess the incidence of sedation failure. From February 2021 to September 2021, children who underwent procedural sedation were retrospectively enrolled. The children were divided into 4 groups based on age: the infant group (0 to 1 year old), toddler group (1 to 3 years old), preschool group (3 to 6 years old), and school-age group (6 to 12 years old). Two-mcg/kg intranasal dexmedetomidine and 0.5-mg/kg oral midazolam were used for sedation. The sedation success rate after rescue, sedation success rate, onset time of sedation, and the sedation time were recorded. The incidence of adverse events and the risk factors for sedation failure were also analyzed. A total of 4758 patients were identified. After exclusion, 3149 patients were ultimately enrolled. The combination of 2-mcg/kg intranasal dexmedetomidine and 0.5-mg/kg oral midazolam resulted in a total success rate of 99.7% and a sedation success rate of 91.4%. The sedation success rate varied among the four groups: 90.2% in the infant group, 93.1% in the toddler group, 92.7% in the preschool group, and 78.4% in the school-age group. The sedation success rate was significantly lower in the school-age group compared to the other three groups (*P* < 0.001). The onset time of sedation was shorter in infant (22 min, IQR: 18–28 min, *P* < 0.001) and longer in the school-age group (30 min, IQR: 25–35 min, *P* < 0.05). Additionally, the infants had a longer sedation time (110 min, IQR: 90–135 min, *P* < 0.001) and a higher rate of delayed recovery (27.5%, all *P* < 0.001). The incidence of adverse events was low (4.70%), which bradycardia (2.03%) being the most common. Age (0–1 year and > 6 years), weight, ASA class II, and history of failed sedation were identified as risk factors of sedation failure.

*Conclusion*: Intranasal administration of 2-mcg/kg dexmedetomidine combined with oral administration of 0.5-mg/kg midazolam was found to be efficient and safety for pediatric procedural sedation. Different age groups of children exhibited distinct sedation characteristics, and age was identified as a risk factor affecting the efficacy of sedation.

**Table:** 

**What is Known:** • *Procedural sedation for diagnostic examination is a common practice in children.* • *The combination of dexmedetomidine with midazolam can improve sedative effects.*	
**What is New:** • *The success rate of sedation using a combination of 2-mcg/kg intranasal dexmedetomidine and 0.5-mg/kg oral midazolam was significantly lower in school-age children as compared to infants, toddlers, and preschoolers.* • *The onset time of sedation increased with age, and the sedation time was found to be longer in infant patients.*

**What is Known:**

• *Procedural sedation for diagnostic examination is a common practice in children.*

• *The combination of dexmedetomidine with midazolam can improve sedative effects.*

**What is New:**

• *The success rate of sedation using a combination of 2-mcg/kg intranasal dexmedetomidine and 0.5-mg/kg oral midazolam was significantly lower in school-age children as compared to infants, toddlers, and preschoolers.*

• *The onset time of sedation increased with age, and the sedation time was found to be longer in infant patients.*

## Introduction

Diagnostic examinations with prolonged immobilization postures pose a challenge due to potential fear of the environment and equipment in infants and young children [1]. Therefore, procedural sedation is often necessary for young pediatric patients. Common drugs used for pediatric procedural sedation and analgesia include chloral hydrate and midazolam [2]. However, chloral hydrate has several drawbacks, such as an extended half-life and a notable propensity for nausea and vomiting after administration [3, 4]. On the other hand, midazolam offers advantages such as antianxiety, sedative, anterograde amnesia, and hypnotic activities, making it one of the most widely used sedative agent in children [5]. Nevertheless, the sedative effect of midazolam is poor for children [6]. Studies have demonstrated that combining oral midazolam with other drugs is more effective in achieving a satisfactory level of sedation in children [7, 8].

Dexmedetomidine is a highly selective and specific α2-adrenergic receptor agonist known for its minimal respiratory depressive effect and high safety profile in infants and children [9]. Intranasal administration of dexmedetomidine is less irritating for children and has a similar effect to intravenous administration [10]. Dexmedetomidine stands out for its ability to produce sedation that closely resembles natural sleep. However, patients may wake up due to external stimuli such as noise or motion [11]. The success rate of using dexmedetomidine alone for sedation is relatively low. Some studies have shown that the combination of dexmedetomidine with midazolam can improve sedative effects [12–14]. However, the effectiveness of dexmedetomidine sedation is influenced by factors such as age, weight, and disease [15]. Additionally, there are significant individual differences in the clearance of midazolam in critically ill neonates, infants, children, and adolescents [16]. Therefore, the precise effect and safety of combining oral midazolam with intranasal dexmedetomidine in different age groups of children and the influence of age on sedation effectiveness have yet to be determined.

Our study focused on evaluating the sedative effect and safety of the combing intranasal dexmedetomidine with oral midazolam sedation in children of different ages. Our objective was to establish superior sedation protocols that would enhance the patient experience during medical procedures.

## Methods

### Patient population

This retrospective study was conducted at the Children’s Hospital, Zhejiang University School of Medicine (Hangzhou, China), after receiving approval from the Institutional Review Board (2022-IBR-0289-P-01) and registering in Chinese Clinical Trail Registry (ChiCTR2300067578). Pediatric patients who underwent procedural sedation with intranasal dexmedetomidine and oral midazolam at the Sedation Center of the Children’s Hospital, Zhejiang University School of Medicine from February 2021 to September 2021, were enrolled in the study. Patients were excluded if they received drugs other than a combination of dexmedetomidine and midazolam, if the drug dosage was not 2 µg/kg of dexmedetomidine or 0.5 mg/kg of midazolam, or if there were incomplete recording. The children were divided into four groups based on their age: infant group (0 to 1 year old), toddler group (1 to 3 years old), preschool group (3 to 6 years old), and school-age group (6 to 12 years old).

### Sedation procedure

Patients classified as American Society of Anesthesiology (ASA) III or higher, with a history of allergic reactions to midazolam or dexmedetomidine, liver or kidney dysfunction, severe respiratory obstruction, severe upper respiratory infection (URI), or severe cardiac arrhythmia, were excluded from sedation. After assessing the patient’s overall physical condition, all patients underwent routine fasting. Vital signs were closely monitored, and relevant data was recorded. Interventional procedure such as peripheral venous cannulation, if needed, was conducted before sedation. All patients were administered 2-mcg/kg dexmedetomidine (100 mcg/mL) intranasally and 0.5-mg/kg midazolam (5 mg/mL) orally by a sedative nurse 40 min before the examination. The depth of sedation was assessed using the Modified Observer’s Assessment of Alertness and Sedation (MOAA/S) score. A MOAA/S score less than or equal to 2 points indicated adequate sedation depth. After achieving sufficient sedation, the patient was transported to the examination facility. If the MOAA/S score was still more than 2 points after 40 min or if the patient woke up during the examination (evidenced by body twisting and inability to continue the examination), the sedative nurse would administer a half dose of the original drug or injected 2-mg/kg propofol intravenously for rescue sedation. The examination would continue when the MOAA/S score was less than or equal to 2 points. If the examination could not be completed even after the remediation, it would be canceled and rescheduled. Diagnostic examinations include magnetic resonance imaging (MRI), contrast-enhanced MRI, computer tomography (CT), contrast-enhanced CT, echocardiography, auditory brainstem response, or other procedures. MRI, especially the contrast-enhanced MRI, typically takes a longer time. A plain MRI scan requires about 15 min, while a contrast-enhanced MRI scan requires nearly 20 min.

After completing the examination, the patient was admitted to the recovery room. Children awoke spontaneously without stimulation. The awakening time was defined as the Steward awakening score reaching 4 points. Children were allowed to be discharged if the Steward awakening score was more than 5 points, vital signs were stable, and there were no adverse reactions after eating. A nurse completed a telephone follow-up with all children within 24 h. The anesthesiologist was responsible for the assessment, management, and documenting adverse events. The patients’ vital signs, such as heart rate (HR), blood pressure (BP), pulse oxygen saturation (SpO_2_), and MOAA/S score, were recorded throughout the entire sedation process. The Steward awakening score was evaluated every 30 min during the recovery from sedation.

### Data collection and definitions

Data collection included age, weight, gender, ASA class score, type of examination, primary diagnosis, sedation success rate after rescue, sedation success rate, onset time of sedation, sedation time, and adverse events.

Sedation success was defined as the successful completion of the procedure using a single dose of 2-mcg/kg intranasal dexmedetomidine and 0.5-mg/kg oral midazolam without the need for rescue drugs. The sedation success after rescue was defined as the successful completion of examination using any sedative methods, with or without the use of rescue drugs if necessary. The onset time of sedation was defined as the time from drug administration to the achievement of a satisfactory level of sedation (MOAA/S ≤ 2). Sedation time was defined as the duration from reaching sufficient sedation to awakening. Delayed awakening was defined as sedation recovery time longer than 120 min. Procedure failure was defined as the inability to continue the examination even after administering rescue drugs.

Adverse events include bradycardia (heart rate decrease > 20% of pre-sedation value), hypoxemia (peripheral capillary blood oxygen saturation (SpO_2_) reduction to below 90% or a reduction more than 10% of the baseline value in children with cyanotic congenital heart disease), hypotension (systolic blood pressure decrease of more than 20% from the normal baseline value), hypertension (systolic blood pressure increased more than 20% from the normal baseline value), anaphylaxis, nausea and vomiting, drowsiness, and irritability within 24 h after sedation.

### Statistical analysis

Quantitative data were presented as mean ± standard deviation (SD) or median (interquartile ranges, IRQs). Categorical data were reported as numbers and percentages (*n*, %). We compared the four groups using the Chi-square test or Kruskal-Wallis H test. A *P*-value < 0.05 was considered for statistically significant, while a *P-*value < 0.0083 was defined as statistically significant after Bonferroni correction for comparison among groups in categorical data. Risk factors were initially analyzed using univariate analysis. Then, binary logistic regression (forward, LR, entry level of 0.05, and an exclusion level of 0.1) was used to determine the risk factors for sedation failure. All statistical tests were two-sided and performed using SPSS 20.0 software.

## Results

### Patient characteristic and sedation condition

**Fig. 1:**
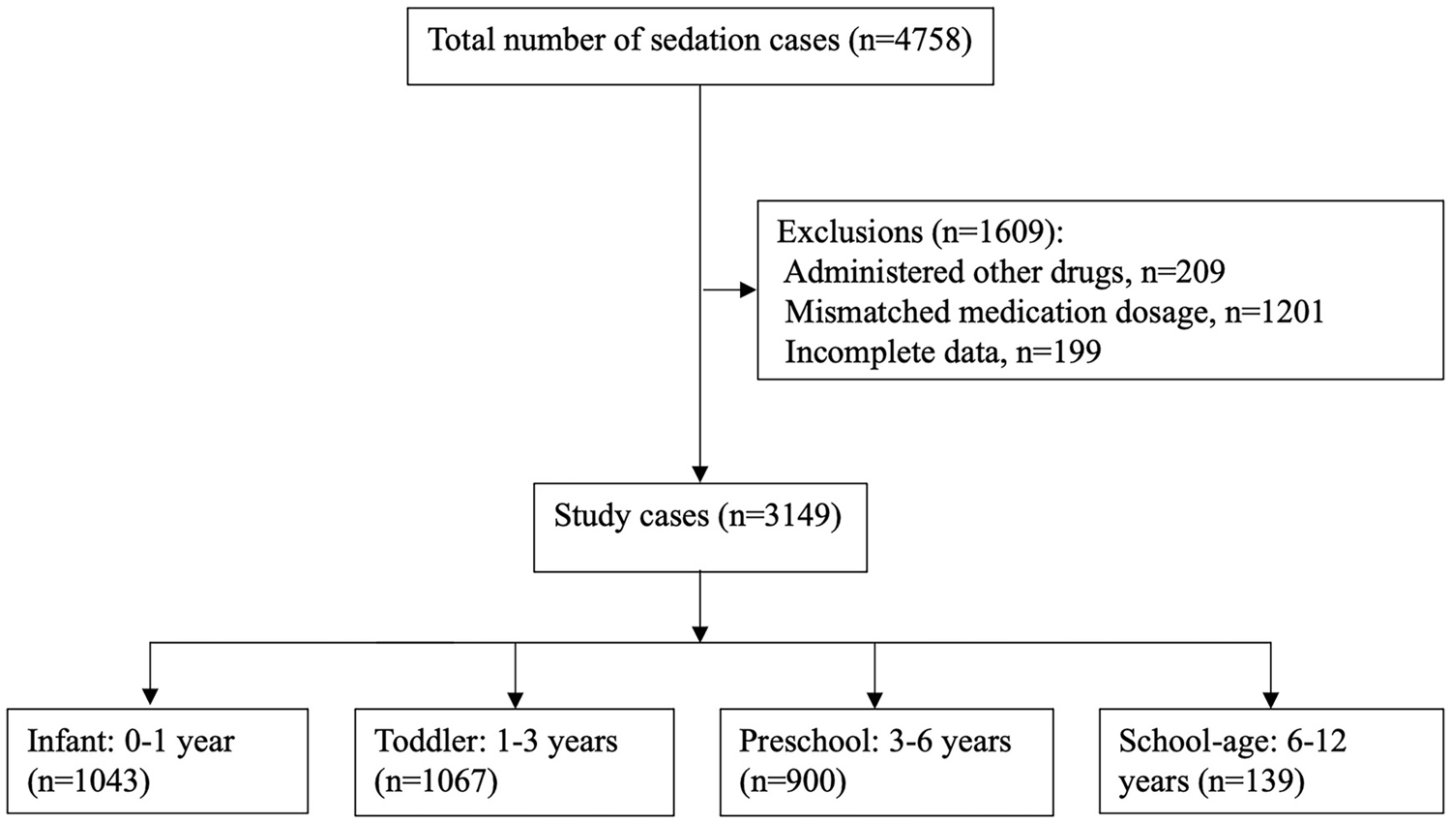
Flow diagram of included patients

Between February 2021 and September 2021, a total of 3149 cases underwent procedural sedation using a combination of 2-mcg/kg intranasal dexmedetomidine and 0.5-mg/kg oral midazolam. The cases were divided into four distinct age groups: infants (0–1 year old) with 1043 cases, toddlers (1–3 years old) with 1067 cases, preschoolers (3–6 years old) with 900 cases, and school-age children (6–12 years old) with 139 cases. The flowchart of the study is presented in Fig. 1. Among the cases, 1945 cases (61.8%) were boys and 1204 cases (38.2%) were girls. The most common diagnostic examination method was magnetic resonance imaging (2988 cases or 94.9%), followed by computer tomography (124 cases or 3.9%). Echocardiography was used in 23 cases (0.7%), auditory brainstem response in 10 cases (0.3%), and four other diagnostic methods were used in the remaining 4 cases (0.1%). Table 1 provides the characteristics and details of the four age groups. The median onset time of sedation was 25 min (IQR: 20–30 min), and the median sedation time was 104 min (IQR: 85–120 min) for all patients, as shown in Table 2.

**Table 1:** Summary of patient characteristics and type of procedure, grouped by age

	**All patients**	**Infant**	**Toddler**	**Preschool**	**School-age**
Characteristics	**(***n*** = 3149)**	**(***n*** = 1043)**	**(***n*** = 1067)**	**(***n*** = 900)**	**(***n*** = 139)**
Age (y)	1.5 (0.7–3.0)	0.4 (0.3–0.7)	1.5 (1.0–2.0)	4.0 (3.0–4.0)	6.2 (6.0–7.4)
Weight (kg)	12.0 (8.6–15.5)	7.8 (6.5–9.0)	12.0 (10.5–13.5)	16.5 (15.0–19.0)	23.0 (20.0–27.0)
Gender (male)	1945 (61.8)	597 (57.2)	666 (62.4)	582 (64.7)	99 (71.2)
ASA class					
I	2549 (80.9)	805 (77.2)	861 (80.7)	769 (85.4)	114 (82.0)
II	600 (19.1)	238 (22.8)	206 (19.3)	131 (14.6)	25 (18.0)
Type of procedure					
MRI	2765 (87.8)	953 (91.4)	907 (85.0)	777 (86.3)	128 (92.1)
Contrast-enhanced MRI	223 (7.1)	39 (3.7)	90 (8.4)	87 (9.7)	7 (5.0)
CT	14 (0.4)	3 (0.3)	9 (0.8)	2 (0.2)	0 (0)
Contrast-enhanced CT	110 (3.5)	43 (4.1)	40 (3.7)	27 (3.0)	0 (0)
Echocardiography	23 (0.7)	3 (0.3)	18 (1.7)	2 (0.2)	0 (0)
ABR	10 (0.3)	2 (0.2)	1 (0.1)	3 (0.3)	4 (2.9)
Others	4 (0.1)	0 (0)	2 (0.2)	2 (0.2)	0 (0)

Data are show as *n* (%) or median (IQR)

*ASA* American Society of Anesthesiologists, *MRI* magnetic resonance imaging, *CT* computer tomography, *ABR* auditory brainstem response

**Table 2:** Sedation success rate and sedation time in different age groups

Group	All patients (*n* = 3149)	Infant (*n* = 1043)	Toddler (*n* = 1067)	Preschool (*n* = 900)	School-age (*n* = 139)	*P*
Sedation success after rescue, *n* (%)	3139 (99.7)	1038 (99.5)	1064 (99.7)	898 (99.8)	139 (100)	0.657
Sedation success, *n* (%)	2877 (91.4)	941 (90.2)	993 (93.1)	834 (92.7)	109 (78.4)	< 0.001^a^
Onset time, min	25 (20–30)	22 (18–28)	25 (20–30)	25 (20–30)	30 (25–35)	< 0.001^b^
Sedation time, min	104 (85–120)	110 (90–135)	100 (80–115)	100 (85–115)	105 (75–120)	< 0.001^c^
Delayed awakening, *n* (%)	589 (18.7)	287 (27.5)	139 (13.0)	134 (14.9)	29 (20.9)	< 0.001^d^

Data are show as *n* (%) or median (IQR)

a, d: Using the chi-square test to compare the success rate of sedation and delayed awakening, a Bonferroni-adjusted *P*-value < 0.0083 is defined as statistically significant for inter-group comparisons in categorical data

b, c: Using Kruskal–Wallis H test to pairwise compare the onset time and sedation time pairwise. The adjusted *P*-value < 0.05 is defined as statistically significant

^a^Adjusted* P* < 0.001 when comparing age group school-age to age groups infant, toddler, and preschool

^b^Adjusted *P* < 0.001 when comparing age group infant to age groups toddler, preschool, and school-age. Adjusted *P* = 0.004 when comparing age group toddler to age group school-age. Adjusted *P* = 0.005 when comparing age group preschool to age group school-age

^c^Adjusted *P* < 0.001 when comparing age group infant to age groups toddler and preschool. Adjusted *P* = 0.006 when comparing age group infant to age group school-age

^d^*P* < 0.001 when comparing age group infant to age groups toddler and preschool

### Sedation characteristic in different age

The sedation success rates were compared pairwise among the different age groups. The success rate for infants was 90.2%, for toddlers was 93.1%, for preschoolers was 92.7%, and for school-age children was only 78.4%. There was a significant difference in the sedation success rate between school-age children and the other three groups (all *P* < 0.001), as indicated in Table 2. There was no significant difference in sedation success rates after rescue among these four groups. The onset time was significantly shorter in the infant group (22 min, IQR: 18–28 min, *P* < 0.001) and longer in the school-age group (30 min, IQR: 25–35 min, *P* < 0.05), but there was no significant difference between the toddler group and the preschool group. The infant group had a significantly longer sedation time (110 min, IQR: 90–135 min, *P* < 0.001) compared to the other groups. Delayed awakening was more common in infant group (27.5%, *P* < 0.001).

### Risk factors of failed sedation

Patient characteristics and comorbidities were evaluated to identify potential factors associated with sedation failure. Table 3 presents the potential risk factors that were analyzed. Through univariate analysis, five potential risk factors significantly associated with sedation failure were identified. Subsequently, binary logistic regression was used to analyze the independent risk factors. Table 4 presents the odds ratio (OR) values and 95% confidence intervals (CI) for each of the identified risk factors: age between 0 and 1 year old (OR 1.788, 95% CI 1.259–2.539, *P* = 0.001), age older than 6 years (OR 2.173, 95% CI 1.227–3.848, *P* = 0.008), weight (OR 1.048, 95% CI 1.014–1.084, *P* = 0.005), ASA class II versus I (OR 1.350, 95% CI 1.001–1.820, *P* = 0.049), and history of failed sedation (OR 1.634, 95% CI 1.254–2.129, *P* < 0.001).

**Table 3:** Univariate analysis for failed sedation

	Number of patients	Number (%) of patients with failed sedation	*P*-value
Age (y)			< 0.001
0–1	1043	102 (9.8)	
1–3	1067	74 (6.9)	
3–6	900	66 (7.3)	
6–12	139	30 (21.6)	
ASA class			0.009
I	2549	204 (8.0)	
II	600	68 (11.3)	
History of failed sedation	945	111 (11.7)	< 0.001
History of surgery	758	77 (10.2)	0.087
URIs			
Running nose	133	12 (9.0)	0.872
Cough	235	20 (8.5)	0.943
Congenital heart disease	278	32 (11.5)	0.074
Growth retardation	431	34 (7.9)	0.550
Weight (kg)	12 (8.6–15.5)	11.25 (8–17)	0.046

Data are show as *n* (%) or median (IQR)

*URI* upper respiratory infection

**Table 4:** Risk factors for failed sedation

Variable	OR	95% CI	*P*-value
Age (0–1 year)	1.788	1.259–2.539	0.001
Age (> 6 year)	2.173	1.227–3.848	0.008
Weight	1.048	1.014–1.084	0.005
ASA grade II	1.350	1.001–1.820	0.049
History of failed sedation	1.634	1.254–2.129	< 0.001

*ASA* American Society of Anesthesiologists

### Major adverse events

**Fig. 2:**
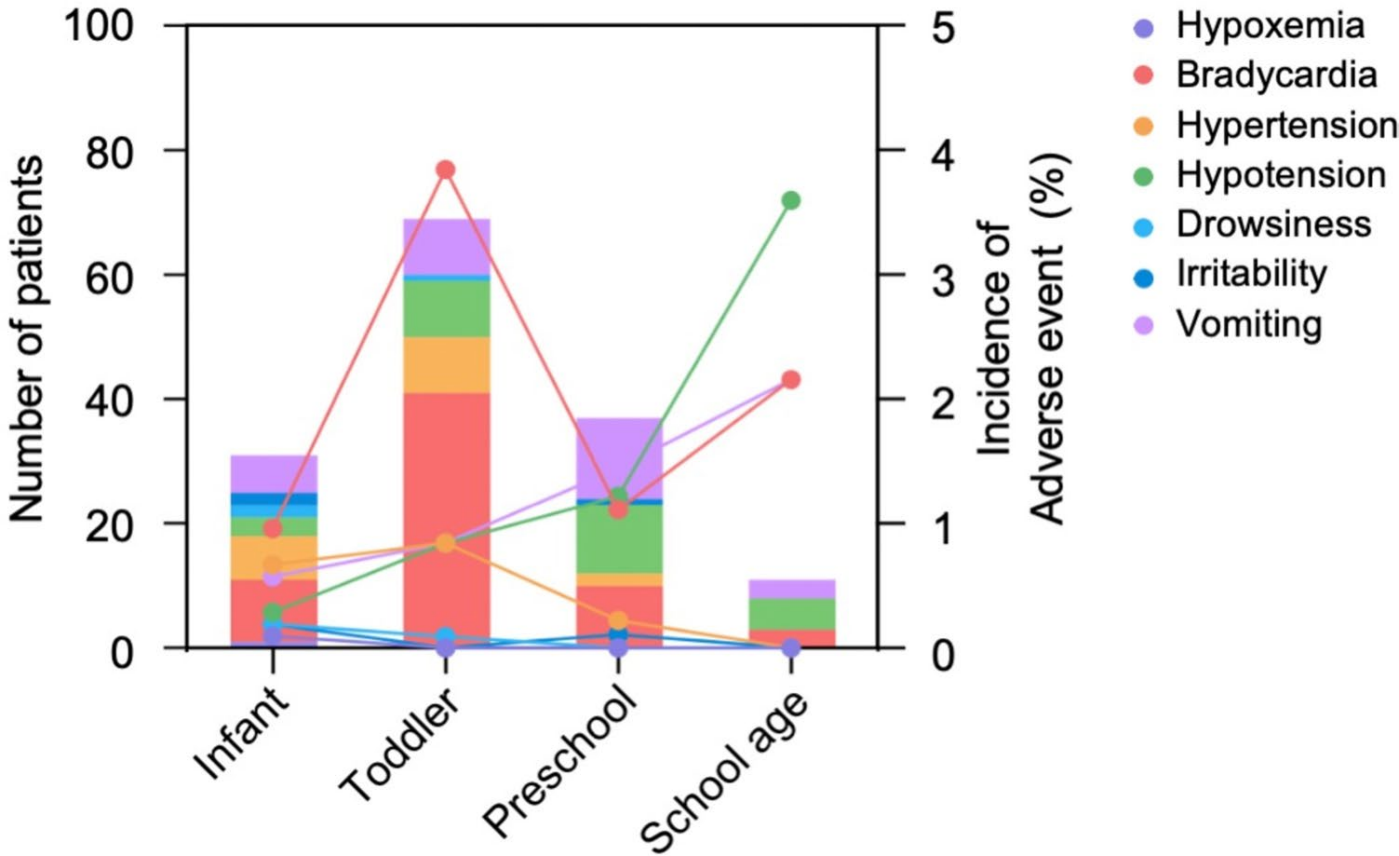
Major adverse events of sedation

A total of 148 (4.70%) major adverse events were reported. The most frequently observed adverse event was bradycardia, which occurred in 64 (2.03%) cases. Nausea and vomiting were reported in 31 (0.98%) cases, followed by hypotension in 28 (0.89%) cases, hypertension in 18 (0.57%) cases, drowsiness in 3 (0.10%) cases, irritability in 3 (0.10%) cases, and hypoxemia in 1 (0.03%) case. The incidence of bradycardia was highest in the infant group (3.8%), while the highest incidence of hypoxemia was observed in the school-age group (3.6%). Figure 2 provides more specific details of adverse events in each age group.

## Discussion

Our study revealed higher sedation success rates after rescue and sedation success rates compared to previous studies [13, 17] using 2-mcg/kg intranasal dexmedetomidine combined with 0.5-mg/kg oral midazolam. Different age groups exhibited varying sedation characteristics. Children younger than 1 year and older than 6 years, as well as weight, ASA class II, and history of sedation, were identified as key factors associated with sedation failure.

In our study, the sedation success rate in school-age children was significantly lower compared to the other age groups. This could be attributed to the higher weight of school-age children, which required a larger volume of dexmedetomidine fluid. However, absorbing a large volume of liquid in a short time may be challenging due to the limited nasal mucosal area. As a result, some of the fluid may flow into the pharyngeal cavity through the back nostrils. The bioavailability of dexmedetomidine through orally administration is poor (16%) due to extensive first-pass elimination effect [18]. Consequently, the sedation success rate is lower in school-age children. Uusalo et al. [19] conducted a study with children aged 5 months to 12 years, using intranasal dexmedetomidine as the sole sedative drug for MRI scans. Almost all patients required additional sedation drugs. This might be due to the dosages used in the study exceeding the recommended intranasal dosing, which necessitated a more concentrated dexmedetomidine solution. In further studies, it would be worth exploring fractional instillation to enhance absorption from the nasal mucosa and increase the sedation success rate. Shin et al. [20] conducted a study and found that children under the age of 3 who receive intravenous injection of midazolam are at risk of paradoxical reactions. Furthermore, most of the contradictory reactions to midazolam are caused by intravenous injection and occur shortly after administration [21]; paradoxical reactions of midazolam are less likely to affect the sedation success rate in school-age children.

This study also observed that the onset time of sedation was shorter in infant group and longer in the school-age group. This finding is similar to a recent study that investigated the use of intranasal dexmedetomidine and buccal midazolam, indicating that the onset time of sedation increased with age [22]. Wang et al. [23] found that an increase in body weight led to later occurrence of maximum plasma concentration, as well as a higher concentration. Uusalo et al. [19] assessed the pharmacokinetics of intranasal dexmedetomidine in pediatric patients aged between 5 months and 11 years old who underwent MRI. They found a negative correlation between age and the peak plasma concentration (C_max_) corrected for dosage and the area under the concentration-time curve corrected for dosage.

Li et al. [24] established an evidence-based dosing regimen using 2-mcg/kg of dexmedetomidine, which achieved the desired threshold of mild to moderate sedation lasting up to 2 h. Similar to our study, which recorded a median sedation time of 105 min in infants and toddlers aged between 0 and 3 years old, these findings suggest a longer duration of sedation in infants, possibly due to the immaturity of the liver’s cytochrome P450 enzyme system. Tateishi et al. [25] compared the expression of 9 different cytochrome P450 (CYP) isoenzymes in the liver between infancy and post-infancy and found higher expression of CPY 1A2, 2B6, and 2C8 in children over 1 year old. Additionally, Hughes et al. [26] estimated the plasma clearance of midazolam in critically ill pediatric patients and described higher plasma clearance in children 3 years and older compared to in infants and children up to 2 years old. These factors may contribute to the higher incidence of delayed awakening in the infant group.

Determining risk factors for failed sedation is crucial for establishing the safety and efficacy of the combination of intranasal dexmedetomidine with oral midazolam for noninvasive procedures in pediatric patients outside of the operating room. Previous studies [27–29] analyzing risk factors for failed sedation in children have shown that history of previous sedation failure, higher ASA score, higher weight, and older age increase the likelihood of sedation failure, which is consistent with our results. Li et al. [22] identified a history of sedation failure as the sole risk factor for sedation failure. On the other hand, Grunwell et al. [28] found that upper respiratory infection was a significant risk factor for failed sedation, whereas our results did not observe a significant effect of URI on sedation failure. This difference may be attributed to the relatively mild symptoms of URI in the children in our study.

Baseline measurements of heart rate, blood pressure, respiratory rate, and pulse oximetry in pediatric patients before sedation may not accurately reflect the actual resting baseline due to factors such as anxiety and crying [30, 31]. In our study, we defined bradycardia and blood pressure changes using normal values at different ages as the baseline [32]. We observed a higher incidence of bradycardia in the toddler group (3.8%) and slightly higher incidence of hypotension in the school-age group (3.6%). However, both rates were lower than those reported in previous studies [13], and no interventions were required. These side effects resolved spontaneously with patient awakening. Overall, our study demonstrated a low incidence of blood pressure changes, pulse oximeter changes, vomiting, drowsiness, and irritability, indicating the high safety of combining intranasal dexmedetomidine with oral midazolam.

Several limitations should be considered when interpreting the results in this study. Firstly, as with most retrospective studies, there may be limitations in the accuracy of patient information collected and estimated by different doctors. Secondly, the number of cases in the school-age group was significantly lower than the other three groups, which may introduce statistical errors. Thirdly, this study includes several different diagnostic procedures, which needed different depth and duration of sedation. This variability may potentially impact the ultimate outcome of the sedation procedure.

## Conclusions

To conclusion, the combination of 2-mcg/kg intranasal dexmedetomidine with 0.5-mg/kg oral midazolam is a safe and effective sedation procedure for noninvasive examinations in pediatric patients. Different age groups exhibit varying sedation characteristics and pose different risks for sedation failure. Further studies using different dosages regimens are needed to ensure optimal sedation for children of different ages.

## Data Availability

Available upon request to the corresponding author.

## Abbreviations

ASA: American Society of Anesthesiology
BP: Blood pressure
CI: Confidence intervals
CT: Computer tomography
CYP: Cytochrome P450
HR: Heart rate
IQR: Interquartile range
MOAA/S: Modified Observer’s Assessment of Alertness and Sedation
MRI: Magnetic resonance imaging
OR: Odds ratio
URI: Upper respiratory infection
